# Exponential increase in the number of prescriptions for ADHD medication between 2012 and 2022 for in Poland.

**DOI:** 10.1192/j.eurpsy.2024.718

**Published:** 2024-08-27

**Authors:** M. Rzeszutek, L. Konowałek

**Affiliations:** ^1^Department of Child Psychiatry, Medical University of Warsaw, Warsaw, Poland

## Abstract

**Introduction:**

ADHD medication prescribing trends are increasing in North America and both Northern and Western Europe (Raman, Sudha R *et al. Lancet Psychiatry.* 2018;5(10):824-835). Methylphenidate and atomoxetine are two substances available for use in ADHD in children and adolescents in Poland. To our knowledge, there is the lack of data on prescription trends for Poland and Middle-Eastern Europe.

**Objectives:**

The aim of the study is to estimate the increase in the total number of prescriptions for methylphenidate and atomoxetine and factors influencing it, like the impact of the proportion of prescriptions for women and for people aged 18-24 on.

**Methods:**

Methylphenidate and atomoxetine prescription data for the period between 2012-2022 and for patients aged 5-59 were obtained from e-Health Centre, which contains data on prescribed medications in Poland. We conducted a series of linear regression models to explore the relationship between the number of prescriptions as the dependent variable and calendar year as the independent variable. Additionally, we considered two more variables: Percentage of prescriptions for women and percentage of prescriptions people aged 18 – 24. Further, we decided to run a mediation analysis to see whether the effect of calendar year was mediated by percentage of women.

**Results:**

We analyzed data on 925,536 prescriptions for methylphenidate and atomoxetine.

The model demonstrates a robust and statistically significant ability to explain the variance in the log-transformed dependent variable (R² = 0.98, F(2, 8) = 201.14, p < 0.001). The model’s intercept, corresponding to calendar year = 0 and percentage of prescriptions for women = 0, is estimated at -93.95, with a 95% confidence interval of [-152.74, -35.15]. The t-statistic for the intercept is -3.68, and the associated p-value is 0.006, demonstrating its statistical significance.

Within this model, the effects of the independent variables are as follows:

1. Calendar year (β=0.05, t=4.07, IC95%: (0.02, 0.08), p<0,004)
2. Percentage of prescriptions for women (β=0.06, t=4.18, IC95%: (0.02, 0.09), p<0,003)

The inclusion of the percentage of prescription for people aged 18-24 doesn’t improve the model’s ability to explain the variation in the number of prescriptions.

Mediation analysis showed that the indirect effect of percentage of prescriptions for women were significant.

**Conclusions:**

These results provide robust evidence for the predictive power of the model, with both calendar year and percentage of women emerging as statistically significant and positively associated with the log- transformed dependent variable.

Between 2012 and 2022, the number of prescriptions for methylphenidate and atomoxetine increased exponentially in Poland. The percentage of prescriptions for women significantly contributed to the increase in the total number of prescriptions for methylphenidate and atomoxetine in Poland.

**Disclosure of Interest:**

None Declared

